# 以反复自发性肝破裂为表现的系统性轻链型淀粉样变性一例

**DOI:** 10.3760/cma.j.issn.0253-2727.2021.11.015

**Published:** 2021-11

**Authors:** 峰 智, 园园 王, 燕萍 马, 伟 张, 丽君 宋, 俊敏 陈, 玉萍 魏, 蓉 李, 娟 田, 慎 包

**Affiliations:** 宁夏回族自治区人民医院、西北民族大学第一附属医院血液内科，银川 750021 Department of Hematology, People's Hospital of Ningxia Hui Autonomous Region, First Affiliated Hospital of Northwest Minzu University, Yinchuan 750021, China

患者，女，67岁，因“乏力、纳差11个月，自发性肝破裂3次”在2021年1月13日于我科住院治疗。患者于我科就诊前曾到外院行PET-CT、胃肠镜等检查，均未明确诊断。患者于2020年7月20日无明显诱因突然出现腹痛、腹胀，就诊于当地医院，腹部CT显示肝包膜下血肿并腹腔积液，腹腔穿刺抽出不凝血，查HGB 83 g/L，PLT 24×10^9^/L，急诊行剖腹探查术，诊断为自发性肝破裂，予肝脏修补术。患者分别于2020年10月8日、2020年11月20日再次因自发性肝破裂就诊于外院，腹部CT示：肝脏破裂修补术后，肝脏体积增大，肝包膜下血肿可见，胸腹腔积液。予补液、输血等保守治疗后好转。患者入院时有乏力、纳差、腹痛、腹胀、口干、体重减轻（1年内体重减轻约5 kg）等症状。入院查体：皮肤黏膜颜色苍白，未见黄染及出血。全腹无压痛及反跳痛，肝脏肋缘下约2横指可触及，质韧，脾脏肋缘下未触及。血常规：WBC 5.73×10^9^/L，HGB 77 g/L，PLT 54×10^9^/L；生化常规：白蛋白37.5 g/L，球蛋白 27.6 g/L，总胆红素 22 µmol/L，间接胆红素14 µmol/L，ALP 204 U/L（正常范围50～135 U/L），GGT 68 U/L（正常范围7～45 U/L），β_2_-微球蛋白4.92 mg/L，IgG 14.24 g/L，IgA 1.59 g/L，IgM 0.89 g/L，LDH 231 U/L；凝血功能：纤维蛋白原（FIB）1.58 g/L，纤维蛋白降解产物（FDP）349.61 µg/ml，超敏D-二聚体 53.58 µg/ml，凝血因子Ⅷ、Ⅸ活性正常，凝血因子Ⅹ活性63.6％（正常范围70％～120％）。外周血涂片未见异常，血清铁6 µmol/L，总铁结合力35 µmol/L，铁饱和度16.84％，铁蛋白532 ng/ml，尿蛋白（++），24 h尿蛋白定量387 mg；B型脑钠肽（BNP）4097 pg/ml，肌钙蛋白0.030 ng/ml。直接Coombs试验阴性，CD55、CD59阴性；血清免疫固定电泳阴性，尿免疫固定电泳示κ链阳性，尿游离轻链κ 282.5 mg/L，λ 49.9 mg/L，比值5.66；血清游离轻链κ 153.17 mg/L，λ 29.8 mg/L，比值5.14。腹部B超示肝脏弥漫性增大，肝脏最大长径为16 cm，肝内无占位。心脏超声示：室间隔厚度11 cm，轻度肺动脉高压，射血分数51.9％，心脏钆造影MRI示：左心扩大并左心舒张功能减退，左室心肌少量纤维化，轻度肺动脉高压，少量心包积液，心内膜不均匀增厚并延迟强化。四肢神经肌电检测未见明显异常。骨髓涂片示：浆细胞3％；骨髓活检提示：成熟浆细胞散在小簇分布，骨髓流式细胞术可见1.1％ CD38^+^异常单克隆浆细胞，骨髓核型分析：46，XX[20]。核磁共振全身弥散加权成像未见骨质破坏。唇腺、皮下脂肪、骨髓组织活检刚果红染色结果均为阳性，偏振光下呈现双折光苹果绿。于唇腺活检组织刚果红阳性部位行显微切割/蛋白质谱分析证实为轻链κ，明确诊断为系统性轻链型淀粉样变性（Mayo 2012分期3期；肝脏、心脏、骨髓、唇腺、皮下脂肪组织受累）。由于患者消瘦明显，予减量的PD方案（硼替佐米1 mg，皮下注射，第1、4、8、11天；地塞米松10 mg/d，静脉滴注，第1～4天，第8～11天）治疗2个疗程，患者精神、食欲明显改善，体重增加约2.5 kg，目前未再发生肝脏破裂出血。治疗后复查血常规示：WBC 8.69×10^9^/L，HGB 93 g/L，PLT 209×10^9^/L；生化常规示：ALP 121 U/L，GGT 43 U/L；凝血功能：FIB 2.73 g/L，FDP 11.74 µg/ml，超敏D-二聚体 2.079 µg/ml；BNP 1925 pg/ml，尿免疫固定电泳阳性，尿游离轻链κ 135.5 mg/L，λ 28.5 mg/ L，血清游离轻链κ 71.3 mg/L，λ 20.6 mg/L。评估血液学疗效为部分缓解，器官疗效评估心脏达缓解标准，肝脏尚未达缓解标准，目前患者仍在定期治疗中。

讨论：系统性轻链型淀粉样变性可累及多种器官及组织，肝脏受累比例为20％～30％。肝脏淀粉样变性常表现为水肿、消化系统症状、体重下降、乏力、腹水等，实验室检查提示ALP、GGT升高而转氨酶、胆红素正常或轻度升高，影像学检查提示肝大，容易漏诊误诊。以反复自发性肝破裂为表现的系统性轻链型淀粉样变性临床罕见，病情凶险，预后差，值得临床医师高度重视。

